# Septicemia in a Neonate following Therapeutic Hypothermia: The Literature Review of Evidence

**DOI:** 10.1155/2013/514232

**Published:** 2013-09-04

**Authors:** Kam Lun Hon, Joshua J. X. Li, Bernadette L. Y. Cheng, Alexander K. C. Leung

**Affiliations:** ^1^Department of Pediatrics, The Chinese University of Hong Kong, Prince of Wales Hospital, 6/F Clinical Sciences Building, Shatin, Hong Kong; ^2^The Chinese University of Hong Kong, Prince of Wales Hospital, Shatin, Hong Kong; ^3^Department of Pediatrics, The University of Calgary, Calgary, AB, Canada T2M 0H5

## Abstract

We report a term neonate with hypoxic ischemic encephalopathy who underwent a 72-hour therapeutic hypothermia. He developed unstable body temperature associated with coagulase negative staphylococcus septicemia 2 weeks later which was promptly treated with intravenous antibiotics and made a good recovery. PubMed (a service of the U.S. National Library of Medicine) was searched for the terms “therapeutic hypothermia” and “septicemia,” with limits activated (humans, English, age 0–18 years). There were only 6 randomized controlled trials, 1 non-randomized controlled trial, 1 retrospective cohort, and 1 case-control trial, which showed no definite evidence of increased risk of septicemia or neutrophil dysfunction in infants following hypothermia therapy.

## 1. Introduction

Hypothermia has been established as an efficacious treatment modality for neonatal hypoxic ischemic encephalopathy [1–8]. Nevertheless, there has been a concern that hypothermia may predispose a neonate to infection during and following hypothermia [9]. We report a term neonate with hypoxic ischemic encephalopathy who underwent therapeutic hypothermia. He developed unstable body temperature associated with coagulase negative staphylococcus septicemia 2 weeks later. Literature review was performed to evaluate if therapeutic hypothermia may predispose a neonate to septicemia.

## 2. Case

Labor was induced for suboptimal cardiotocography and fetal scalp blood sampling (low pH 7.007), and a primigravida gestation 37-weeks and 2-day male, weighing 3 kg, was delivered by emergency caesarean for fetal distress and moderate meconium stained liquor. Antenatal history was unremarkable apart from gestational diabetes managed with diet. The baby was born flaccid and cyanosed, and meconium was aspirated by endotracheal tube suction. His heart rate increased from 80/min to 120/min after one minute of face-mask bagging. Cord blood pH was 6.8 with Apgar scores 2 and 5 at 1 and 5 min, respectively. There was some grunting and chest insucking. He was transferred to the neonatal intensive care unit (NICU) for the management of birth asphyxia. He underwent a 72-hour duration of therapeutic hypothermia (33-34°C) and made an uneventful recovery. There was no evidence of encephalopathy throughout the treatment period. On the eleventh day of life, the baby developed a febrile episode which was treated with a course of vancomycin and gentamicin. The C-reactive protein was raised to 26.7 (highest), and blood culture yielded coagulase negative *Staphylococcus* species (CNSS) which was sensitive to vancomycin. Cerebrospinal fluid and urine culture was negative. Serial white cell count and differentials were within normal limits during the course of hypothermia and the febrile episode. He was discharged home after 5 weeks. Normal growth and development were noted at 1, 3, and 6-month followups.

## 3. Discussion

In order to address the clinical question if therapeutic hypothermia predisposes pediatric patients to the risk of sepsis, PubMed was searched using “infection” and “therapeutic hypothermia” as keywords. Results were limited to the range from 0 to 18 years and human studies. 112 results were retrieved. 107 articles were excluded as they were irrelevant, written in non-English language, nonpediatric study population, noniatrogenic hypothermia, intraoperative hypothermia instead of therapeutic hypothermia, or infection not measured as an outcome. References of the remaining 4 articles were reviewed, and 5 more articles were included as citations in the references (Table 1).

Studies include randomized controlled trials (*n* = 6), with one article reporting two trials conducted simultaneously by the same authors [11], nonrandomized controlled trials (*n* = 1), retrospective cohort (*n* = 1), and case-control trials (*n* = 1). In the case-control trial retrieved, there was no incidence of infection in the control arm for odds ratio calculation (Table 1) [9]. The types of infection included in the studies reviewed included sepsis [2, 9], pneumonia [2, 9], urinary tract infection [2], and positive cultures [12, 15]. 

As the *P* values in some of the studies were not specified, they were calculated by SPSS v20.0, using Fisher's exact test (Table 2). In all of the articles reviewed, 4 studies [11, 13–15] reported a relative risk less than 1 for developing infection after therapeutic hypothermia, and one showed a relative risk greater than 1 [12], but none reached statistical significance. No study showed if posthypothermia patients are at risk of certain infections, including any bacterial, fungal, or viral pathogens. Nor was there any current evidence to indicate if patients are prone to infections during or immediately following hypothermia. Therefore, our case of CNSS septicemia may be an incidental finding of septicemia in a hospitalized neonate.

 Neutrophils may be relevant in bacterial infections. Two studies [2, 10] reported that there was no difference in the white cell counts between the hypothermia and control groups (normothermic group), in contrast to in vitro studies which show the impairment of neutrophil function at low temperatures [9]. 

To conclude, no published literature demonstrates that therapeutic hypothermia would affect a neonate's neutrophil number or function in vivo or predispose the patient to septicemia. Nor was there any evidence to suggest that these neonates were susceptible to a certain class of pathogens during or following hypothermia therapy in the neonatal period. Our literature review suggests that therapeutic hypothermia does not predispose a child to any subsequent risk of infection. Further research is required to establish the long-term complications of therapeutic hypothermia.

## Figures and Tables

**Table 1:** Publications of therapeutic hypothermia reviewed.

	Population	Study type	Reason for hypothermia	Remarks
Hall et al., 2010 [10]	26–30 weeks	Nonrandomized controlled trial	Necrotizing enterocolitis	Neutrophil count measured
Adelson et al., 2005 [11]	0–13 years	Randomized controlled trial	Traumatic brain injury	Children also recruited
Adelson et al., 2005 [11]	0–17 years	Randomized controlled trial	Traumatic brain injury	Children and adolescence also recruited
Eicher et al., 2005 [2]	≥35 weeks	Randomized controlled trial	Neonatal encephalopathy	Neutrophil counts measured; infection stratified according to site (UTI, pneumonia, and sepsis)
Gluckman, 2005 [12]	≥37 weeks	Randomized controlled trial	Neonatal encephalopathy	Large scale trial (*n* > 200)
Shankaran, 2005 [13]	≥36 weeks	Randomized controlled trial	Hypoxic ischemic encephalopathy	Large scale trial (*n* > 200); septicemia measured
Akisu et al., 2003 [14]	≥37 weeks	Randomized controlled trial	Perinatal asphyxia	Location of infection not specified
Gunn et al., 1998 [15]	≥37 weeks	Randomized controlled trial	Perinatal asphyxia	No infection reported in hypothermic group
Clardy et al., 1985 [9]	Pediatric	Case-control trial	Hypoxic ischemic encephalopathy	Neutrophil studies performed

**Table 2:** Relative risk and *P* value.

	Relative risk (95% C.I.)	*P* value
Hall et al., 2010 [10]	Not reported	
Adelson et al., 2005 [11]	0.80 (0.28–2.26)	0.67
Adelson et al., 2005 [11]	1.00 (0.32–3.17)	1.00
Eicher et al., 2005 [2]	1.00 (0.22–4.58)	1.00
Gluckman, 2005 [12]	1.05 (0.22–5.11)	1.00
Shankaran, 2005 [13]	0.87 (0.27–2.75)	1.00
Akisu et al., 2003 [14]	0.54 (0.05–4.28)	0.59
Gunn et al., 1998 [15]	0.72 (0.05–10.52)	1.00
Clardy et al., 1985 [9]	Cannot be calculated	
